# Community Health-Education Intervention Trial against Human *Taenia solium* Taeniasis/Cysticercosis in Central and Southern Zones of Tanzania

**DOI:** 10.3390/pathogens12070955

**Published:** 2023-07-20

**Authors:** George Makingi, Bernard Ngowi, Ernatus Mkupasi, Christina Wilson, Andrea Sylvia Winkler, Jahashi Nzalawahe, Helena Ngowi

**Affiliations:** 1 The College of Veterinary Medicine and Biomedical Sciences, Sokoine University of Agriculture, Morogoro P.O. Box 3021, Tanzania; 2 Muhimbili Medical Research Centre, National Institute for Medical Research, Dar es Salaam P.O. Box 3436, Tanzania; 3 Mbeya College of Health and Allied Sciences, University of Dar es Salaam, Mbeya P.O. Box 608, Tanzania; 4 Center for Global Health, Department of Neurology, Technical University of Munich, 81675 Munich, Germany; 5 Department of Community Medicine and Global Health, Institute of Health and Society, University of Oslo, 0373 Oslo, Norway

**Keywords:** human, Taeniosis, cysticercosis, education, intervention, neglected diseases

## Abstract

Poor knowledge of human *T. solium* taeniasis/cysticercosis and insufficient sanitary and hygienic practices have been associated with the persistence of human *T. solium* infections in endemic areas. Community health education intervention measures were implemented in 42 villages of Kongwa and Songwe Districts to increase knowledge, improve good practices against infection and reduce incidences of human cysticercosis transmission using a health education package. The health education package comprised of leaflet, poster and a booklet The 42 villages were allocated into intervention group and control group, and each group consisted of 21 villages. Baseline and post-intervention information on social demography, knowledge, safe practices and incidences of human cysticercosis was collected from both village groups. The impact of the intervention was evaluated by comparing changes in knowledge, preventive practices related to human *T. solium* infections and the cumulative incidence of human cysticercosis between intervention and control villages. There was no significant difference in mean knowledge scores and preventive practice mean scores between the control and intervention groups at baseline. However, there were significantly higher knowledge mean scores in the intervention group compared to the control group at one year post-intervention (2.06 ± 1.45 vs. 0.94 ± 1.18, *p* < 0.001). There was no significant difference in the mean practice scores between the intervention and the control group at one year post-intervention (2.49 ± 1.13 vs. 2.40 ± 1.13, *p* = 0.31). Furthermore, there was no significant difference in the prevalence of human *T. solium* cysticercosis between the intervention and the control group at the baseline (1.4% vs. 1.4%, *p* = 0.97) by Ag-Elisa, and at one year post-intervention the cumulative incidence of human cysticercosis was 1.9 and 1.2 per cent in the control and intervention group, respectively. There was no significant difference in the cumulative incidence of human cysticercosis between the intervention and the control group at one year post-intervention (*p* > 0.05). Community health-education intervention is effective at improving the knowledge of human *T. solium* infections. The improvement in preventive practices and reduction in incidences of human cysticercosis are a gradual process, they may require sanitary and hygienic improvement and more time after the intervention to see improved changes. The study recommends a sustainable public health education on *T. solium* infections using the health education package through one health approach.

## 1. Introduction

*Taenia solium*-taeniasis and cysticercosis (TSTC) are diseases mostly endemic in developing countries, associated with poverty, poor hygiene, inadequate sanitation and unrestricted pig movement [1]. Humans can harbour the adult intestinal tapeworm (taeniasis), the sole natural definitive host, acquired by eating raw or undercooked infected pork. Infective eggs are shed by human carriers via the stool and contaminate the environment. Pigs are significant intermediate hosts and are infected by ingestion of these infective eggs (or proglottids), which develop into porcine cysticercosis (PCC). Humans act as intermediate dead-end hosts, where cysts can develop in muscles leading to human cysticercosis (HCC) and upon reaching the central nervous system (CNS) leads to neurocysticercosis (NCC) [2]. Although the manner in which eggs are ingested is not usually known, the tapeworm carriers and close household contacts are at the greatest risk, suggesting that person-to-person spread is important. However, the contamination of food and water also occurs. Thus, HCC can occur in individuals who do not raise pigs or consume pork [3]. The burden of *T. solium* infection is mainly due to the public health effects of NCC and economic losses due to associated treatment. The World Health Organization (WHO) estimated *Taenia* infections to have infected approximately 50 million people annually and to kill 50,000 [1]. The infections are endemic in many countries of sub–Saharan Africa (SSA), Asia and Latin America [4,5].

In most endemic areas, the infection and disease remain uncontrolled because of a lack of information and awareness about the extent of the problem and the absence of suitable diagnostic tools and intervention strategies adapted to the local situation [6]. Recent epidemiologic studies carried out in some areas in Tanzania revealed that significant cases of human *T. solium* infections of up to 17 per cent of cases of HCC have been reported in rural and urban populations [7,8], and identified poor knowledge and risky practices to be associated with transmission [7,9,10]. In Tanzania, the number of disability-adjusted life years (DALYs) per thousand person years for NCC-associated epilepsy was estimated to be 0.7 for the year 2012. The annual number of NCC-associated epilepsy incident cases and deaths were 17,853 and 212, respectively [11]. Additionally, around five million USD were spent due to NCC-associated epilepsy, comprising direct and indirect costs due to hospitalizations, visits to the hospitals/doctors and the cost of drugs/medicines [1]. Control measures recommended to combat this disease include improvements in hygiene, sanitation, pig management, mass taeniacidal chemotherapy and health education [12]. However, there are limited community studies on the evaluation of such interventions in endemic countries, Tanzania included, where only one study has been carried out to assess the impact of health education on the prevalence or incidence of *T. solium* infections. The study observed a decrease in the incidence of PCC about one year after a health-education intervention, as assessed by Ag-ELISA using sentinel pigs. In this study, the increase in knowledge did not significantly change observed behaviour [13]. Health education is the cornerstone of health promotion and has been defined as the lifelong process by which individuals acquire knowledge, attitudes and behaviour that promote health and foster wise decisions for solving personal, family and community health problems [14]. Furthermore, health educational intervention is highly encouraged because it might lead to permanent changes towards controlling and eventually eliminating the infections. Based on a study performed in the Mbulu, Mpwapwa and Mbinga Districts in Tanzania where knowledge regarding TSTC was low, researchers identified certain key messages from the community [15] leading to the development of a community-based health education intervention package for preventing and controlling TSTC.

This study hypothesized that imparting specific health education to the community regarding human TSTC would increase their knowledge, improve their preventive practices against the infection and eventually reduce incidences of HCC in endemic areas. Therefore, the study was designed to evaluate the effectiveness of a locally developed health education trial against human *Taenia solium* taeniasis/cysticercosis in villages of the Kongwa and Songwe Districts representing the central and the southern zones of Tanzania, respectively.

## 2. Materials and Methods

### 2.1. Study Area

The survey was conducted from September 2019 to December 2021 in the villages of the Songwe and Kongwa Districts of Tanzania. The districts were selected purposefully based on known *T. solium* infection endemicity and accessibility [7,16,17,18]. Songwe District is located in the southern highlands of Tanzania with an altitude ranging from 900 to 2750 m above sea level, between latitude 8°11′ to 57°84′ south of the equator and longitude 32°53′ to 41°10′ east of Greenwich Meridian. This district covers an area of 16,070 square kilometres composed of 43 villages. It has an estimated population of 157,089 people within 28,282 households. The main occupational activities include agriculture, mining and other informal sectors. The mean temperature is about 26.5 °C. The district has one rainy season from November to May, which records 750 to 2000 mm per annum [19].

Kongwa District is located in the central zone of Tanzania at a latitude of 5°30′ to 6°00′ south of the equator and longitudes 36°15′ to 36°00′ east of the Greenwich Meridian. Its height ranges between 900 and 1000 m above the main sea level. This district has an area of 4041 square kilometres. It is composed of 87 villages, with an estimated human population of 365,952 composed of 61,914 households [19]. The occupational activities of inhabitants are agriculture and other informal sectors. The mean temperature is about 26.5 °C and the rainy season is from November to April, with an average annual rainfall of 500 to 800 mm [20].

### 2.2. Study Design

The study adopted a cluster-randomised control trial with pre- and post-intervention assessments of study subjects. The study assessed a total of 93 and 43 villages in the Kongwa and Songwe Districts, respectively, for their eligibility to participate in the study to include 42 eligible and virtually independent villages as per the previous similar study in the southern zone of the country [13]. Eligibility criteria for a village to participate in the study included pig-keeping activities and the willingness of the community to participate in the study. The eligibility criteria were met by all 136 villages. A list of all villages including their pig population was obtained and each village was assigned a unique number; using Microsoft Excel, a random sampling procedure was performed whereby 42 villages were randomly selected and enrolled in the study.

### 2.3. Sample Size Estimation and Selection of the Household

The sample size estimation was calculated using the formula *n* = Z^2^PQ/L [21], where *n* is the required number of individuals to be enrolled, Z is the Z-score for a given confidence level, P is a known or estimated prevalence, Q = (1 − P), and L the allowable error of estimation. In the current study, 95 per cent was used as the confidence level with an allowable error of estimation of 0.05. *p* = 16 per cent, the estimated prevalence of HCC nearby the study area [7], and d = 0.05, the relative precision. Thus, *n* = 1.96^2^ × 0.16 × (1 − 0.16)/0.05^2^ = 382 households (one person per household). This number was more than doubled to adjust for dropout during the study and multi-stage sampling design effect and, thus, adjusted to = 872 for the two districts. Participating households were randomly selected from 42 villages in the two districts.

Before the beginning of the study, sensitization and mobilization meetings involving researchers and community leaders were conducted, in which the study objectives were explained. A list of all village households who agreed to participate was obtained, and by using Microsoft Excel Window 10 households for inclusion in the survey were randomly selected. Then, the research team visited the selected households with support from the household head, the eligible household representative were identified to participate in the study. The criteria of eligibility were being a permanent household member, aged between 15–60 years and the willingness to participate.

### 2.4. Data Collection

**Figure 1:**
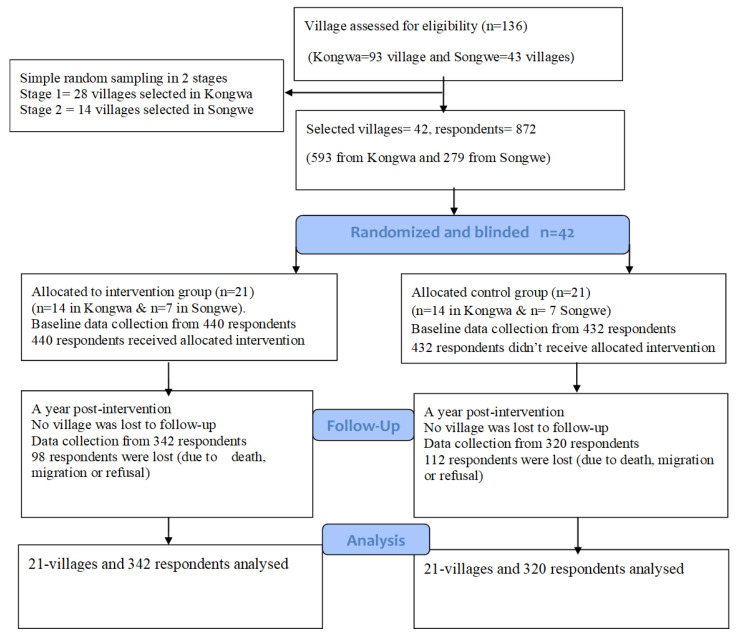
The flow of respondents in a randomized control trial to assess the effect of health-education intervention in the Songwe and Kongwa Districts, Tanzania, 2019–2021.

A total of 42 villages were studied (28 from Kongwa and 14 from Songwe). The baseline study recruited a total of 872 households (513 from Kongwa and 279 from Songwe). During the post-intervention study, a total of 210 households were lost to follow-up (98 from the intervention group and 122 from the control group) due to various reasons, including death and migration to other areas, and some of the respondents refused to continue. The flow of participation is summarized in Figure 1.

#### 2.4.1. Scheme Diagram of Sample Collection

The study collected the data in three key phases: it was first collected between July and September 2019 in the selected villages of the two aforementioned districts during a baseline survey, followed by the health educational intervention which lasted for two months (September and November 2020). The survey was repeated between September and December 2021, a year after the education intervention, whereby data were collected to evaluate the effect of the health education intervention in improving knowledge and preventive practices against human TSTC among community members.

#### 2.4.2. Baseline Survey

The baseline study was conducted between July 2019 and September 2019 to collect information through face-to-face interviews with household heads or representatives. The information was collected using KoboCollect; the application for data collection through KoBoToolBox (Kobo Collect v.1.27.3-3) [22] was downloaded from Google Play Store and installed on Android tablets. The tool was used for the formulation of questionnaires and data collection. The questionnaire was designed based on the WHO guide for developing knowledge, attitude and practices (KAP) surveys [23] and included both closed- and open-ended questions. Two investigators were trained on the use of KoboCollect tool before the commencement of the study. The investigators also pre-tested the feasibility and correctness of the items in the electronic questionnaire. The explored information included social-demographic characteristics, knowledge of human TSTC transmission, symptoms, preventive measures, treatment options and sanitary and hygienic practices as detailed in Table 1. The questionnaire responses were supplemented with direct observation during visits to the households. The questions were phrased according to the local circumstances and Kiswahili language was used to provide the information. After the questionnaire survey, 5 mL venous blood was drawn from all participants who also consented to the serological component of the study. The blood sample was drawn at the cephalic or median cubital vein (median basilic vein) by a medical laboratory technician; the blood was collected in a specific plain blood vacutainer tube. The tubes were placed in a cooler, and left to decant, and the sera were collected and put in two pre-labelled tubes at the end of each day or the following day. The sera were placed in freezers (−20 °C) after the blood draw, and then brought to the laboratory (Public health research laboratory) and kept in a freezer at −20 °C until the analyses were carried out at the College of Veterinary Medicine and Biomedical Sciences laboratories. The serum samples were tested for the presence of excretory circulating antigens of the metacestode of *T. solium* using enzyme-linked immunosorbent assay (Ag-ELISA) [24,25,26]. The test was found to have a sensitivity of 94.4 per cent (95% CI: 80–99%) and a specificity of 98 per cent (95% CI: 90–99%) to detect current infection in a study conducted in Vietnam and Ecuador [27]. With Ag-ELISA, no cross-reactions were observed in sera from patients with confirmed infections with *Schistosoma*, hydatid cysts, *Ascaris*, *Trichuris*, filaria, *Entamoeba*, *Plasmodium* and *Trypanosoma* [28].

#### 2.4.3. Community Health Education Intervention

The intervention consisted of a health-educational package (leaflets, booklets, posters, and a training manual) which was developed during a previous sociological study, conducted in Mbulu, Mpwapwa and Mbinga Districts in Tanzania where knowledge regarding TSTC was low. Researchers identified certain key messages from the community [15] leading to the development of the package for preventing and controlling TSTC. Videos and pictures were locally produced to increase the understanding of both literate and illiterate respondents. The training manuals were made more pictorial than text to enable illiterate respondents to follow the messages. The training was given at the village level whereas the respondents from control villages received only routine community health services. The education intervention was implemented in two phases; the first phase involved 2 days of training for 10–15, with the trainers including livestock/agricultural extension officers, school teachers, health workers and village leaders to serve as local trainers for sustainability purposes. The trainers also suggested improvements to the package to better fit the local situation. The second phase of the intervention was implemented by involving the general public in the intervention group. All respondents were invited to attend 2 days of training at a chosen village-level location. During the training sessions, the trainers with support from the researchers trained respondents using the health-education package. The package was mainly composed of three modules which included a module on knowledge of TSTC and transmission dynamics, a module on hygienic and sanitary practices associated with TSTC, and the last module was on proper pig management. The trainer later distributed a leaflet and a booklet to each household owner. The seminars were conducted in village offices, school classrooms, churches and other available places.

#### 2.4.4. Post-Health Education Intervention Survey

One year after the health education training, the questionnaire survey and direct observations were repeated in all households of the 42 study villages, recruiting the same respondents and assessing the same factors as during the baseline study. A total of 872 and 662 participants from the study villages were surveyed at the baseline and post-intervention, respectively. Again, after the questionnaire survey, the serological survey was repeated. However, this survey involved only participants who were Ag-Elisa-negative at the baseline.

### 2.5. Statistical Analysis

Data were imported from the KoBoCollect toolbox into Microsoft Excel for cleaning. The data were then transferred to IBM Statistical Package for Social Sciences (SPSS version 20.0) for statistical analysis. This analysis involved only households that participated in both the baseline and the post-intervention studies, hereby referred to as “full participants”. The statistical methods used in this work were conducted in three steps. (1) McNemar’s Chi-Square tests were conducted to examine the equilibrium of demographic characteristics (sex, age, location of participant and educational level) at baseline. (2) The frequency of correct answers on the knowledge and preventive practice variables regarding human TSTC and (3) the mean scores on knowledge and preventive practices, each question having an equal weight of knowledge and preventive practices, the mean scores were compared between intervention and control groups through Sharpiro-Wilk test, T-test and Wilcoxon test. In addition, the cumulative incidence of HCC was compared between the intervention and control groups taking into account their baseline prevalence levels.

## 3. Results

### 3.1. Socio-Demographic Characteristics of Respondents at Baseline

The socio-demographic characteristics of participants at baseline in the control and intervention group are presented in Table 1. The total number of participants at baseline and post-intervention was 662, whereby 324 (48.9%) and 338 (51.1%) participants were in the control and intervention groups, respectively. The majority of participants in both groups were males: 230 (71.0%) and 220 (65.1%) from the control and intervention groups, respectively. At baseline, a total of 41 (12.6%) and 48 (14.2%) participants were found to be aware of the infection in the control and intervention groups, respectively. Based on preventive practices, a total of 318 (98.1%) and 334 (98.8%) from the control and intervention groups, respectively, agreed to always observe free-roaming pigs in their community.

### 3.2. Post-Intervention Comparison of Knowledge and Preventive Practices in the Control and Intervention Groups

The proportions of respondents who were able to respond correctly to the given questions following the community health education intervention are shown in Table 2. Regarding awareness of human *T. solium* infections, 60 (18.5%) and 206 (60.9%) of the respondents from the control and intervention groups, respectively, reported to have been aware of the infections. From the intervention group, 159 (47.8%) participants agreed that human epilepsy could be caused by *T. solium* infection compared to 56 (17.4%) participants from the control group. The results also indicated that 115 (34.0%) participants from the intervention group were using official water sources for domestic purposes compared to 75 (23.1%) participants from the control group who were using unofficial water sources (Table 2).

### 3.3. Mean Knowledge and Preventive Practice Scores Regarding Human TSTC at Baseline and Post-Intervention

#### Knowledge Mean Scores

The results showed that there was no significant difference in knowledge mean scores between the control and intervention groups (1.54 ± 1.02 vs. 1.45 ± 0.94, *p* = 0.24) at baseline. However, there was significantly higher knowledge mean scores in the intervention group compared to the control group a year post-intervention (2.06 ± 1.45 vs. 0.94 ± 1.18, *p* < 0.001). The results also showed that there was no significant difference in practice mean scores between the intervention and the control group (3.01 ± 0.06 vs. 2.98 ± 1.01, *p* = 0.678) at baseline. Despite the education intervention given, there was also no significant difference in practice mean scores between the intervention and the control group a year post-intervention (2.49 ± 1.13 vs. 2.40 ± 1.13, *p* = 0.31) (Table 3).

### 3.4. Prevalence of Human Cysticercosis before and Cumulative Incidence after the Health Educational Intervention

The prevalence of HCC showed no significant difference at baseline between the intervention and control groups as measured by Ag-Elisa in the serum sample (Table 4). The comparison of cumulative incidence of the infection between control and intervention villages a year after an intensive health education intervention showed no statistically significant reduction in new infection cases between the control and intervention groups.

## 4. Discussion

This was a randomized, controlled, community-based health-education intervention trial for the control of human *T. solium* infections conducted in an area where the infection is endemic [7,18]. The study utilized health-education intervention as a strategy to increase knowledge, improve preventive practices against human TSTC and ultimately reduce the cumulative incidence of HCC among community members in the selected intervention villages of Kongwa and Songwe District [13,29,30]. The comparison of the pre–post-randomisation change in knowledge, preventive practice and cumulative incidence between the control and intervention groups helped us to freely study the effect of the intervention itself, given that an individual’s behaviour can be altered because the individual knows that he/she is being studied, known as the “Hawthorne effect” [13,31,32]. The observed changes in the outcomes could be attributed to the effect of the intervention delivered to the target villages.

This study and other studies have consistently shown that the knowledge regarding *T. solium* infections is poor, resulting in an increase in infection transmission, and hence recommends specific and targeted education among this at-risk group [7,13,33,34]. At the baseline, this study revealed that most of the participants in intervention and control groups had limited knowledge of human cases of TSTC as the infection was always mentioned in pigs. Additionally, the majority of the participants had limited knowledge of the life cycle of the parasite, believing that it was safe for humans to consume PCC-infected pork. In addition, the majority of the participants did not know that the life cycle of the parasites involves humans and pigs. Considering clinical signs associated with the infection, the majority of the participants did not know that HCC may result in an epileptic condition. Furthermore, most participants did not have knowledge of prevention and control measures against the infection, as they believed that the infection could not be prevented. Additionally, the baseline surveillance revealed the existence of risk practices associated with human TSTC in both groups: as noted, there were massive unrestricted pig movements in both the control and intervention villages. Free-range pig management increases the chances of TSTC transmission in most endemic areas of the country [13,18,33,34]. Additionally, the study observed excessive domestic use of unofficial water sources due to limited official water sources in the study area. This practice is common in most villages of the country where the supply of safe and clean water was not adequate [35], which might have subjected the community to acquiring infectious diseases including HCC. In addition, the majority of participants reported having spotted human faeces around their premises; this practice might cause environmental contamination with various infectious agents including *T. solium* eggs from human *T. solium* carriers. The baseline study also revealed the prevalence of HCC in the study villages, whereby further findings indicated that there was no significant difference in the prevalence between the control and intervention villages. However, the reported prevalence in this study was low compared to previous studies in the country. This might be due to differences in the study design, clinical characteristics of the study population and the diagnostic methods used [7,8]. Health education is the cornerstone of health promotion and has been defined as the lifelong process by which individuals acquire knowledge, attitudes and behaviour that promote health and foster wise decision making for solving personal, family and community health problems [14].

Following the health-education training in the intervention villages, the results of this study showed different mean scores between the control and intervention. There was significantly higher knowledge mean scores in the intervention group compared to the control group, showing an improvement in knowledge among respondents who received the intervention. The intervention resulted in an increase in the knowledge of human TSTC in the intervention group compared to the control group. Likewise, knowledge on the life cycle of the parasite was increased in the intervention group as every participant knew the risk for humans to consume PCC-infected pork. In addition, most of the trained participants agreed that human TSTC could be prevented at the community level after learning of the transmission cycle. This study is in agreement with a previous study that evaluated the effect of health education in improving knowledge and practices related to the transmission of PCC in Mbulu District, northern Tanzania [13], between which the training materials delivery mode. Another community-based study in Mexico reported that an intensive community-participatory educational campaign was associated with an improvement in knowledge and a reduction in PCC [29]; however, this study was not randomised and was conducted in only one community and ignored the dynamics of pig populations in rural areas, and the mode of delivering the training was not the same. Health education training has significantly improved awareness of *T. solium* infections, the transmission cycle of parasites, clinical signs of NCC and the knowledge of preventive measures related to human TSTC. Studies that have evaluated the effect of the health-education intervention on knowledge and preventive practices regarding human TSTC in the country are scarce. However, similar studies in Mbulu District, Tanzania [13,30] showed that an education intervention significantly improved knowledge of PCC; another similar study in Southern India and Mexico [29,36] also showed health education to significantly improve the knowledge of TSTC and the knowledge of the various ways in which NCC is transmitted. Therefore, efforts in providing an effective health-education intervention to the risky group regarding this infection will have a positive impact in improving knowledge and hence controlling the parasite.

However, the given health education training did not show significant improvement in preventive practices in intervention villages. The observed key preventive practices were those closely related to the transmission of human TSTC such as free-range pigs, the possession and use of toilets at each household, water sources for domestic use and hand cleanness after toilet use. This and other studies have shown that preventive practices against TSTC transmission are still suboptimal despite a high level of awareness and availability of preventive services. The finding of this study is consistent with the findings in other studies and demonstrated the effectiveness of health-education interventions in improving preventive practices against TSTC [13,29,37,38]. A lack of improved preventive practices might be attributed to a limited time to determine the effect, individual negligence, poverty, cultural beliefs and the observed inappropriate and insufficient public health services, such as the inadequate safe and clean water supply in most of the endemic areas of the country [10,35,39]. For example, it was observed that a shortage of safe and clean in most villages did not stop some people in the study areas from fetching water from unofficial water sources, a factor that might not be changed in the short run [35,40]. Despite the effort to control contamination by conducting training in the intervention group, contamination in the control group could occur. The health-education intervention resulted in a significant decrease in participants who always observed free-roaming pigs in both the intervention and control groups due to the effect of the intervention and the ‘‘Hawthorne effect.” Furthermore, the observed practices were measured cross-sectionally and might not reflect the practices used most of the time. In consideration of the importance of preventive practices for infection prevention and control as a requirement, the need for information and positive attitude changes towards the disease is also important. Another important component of the present study was the estimation of the cumulative incidence of HCC a year post-intervention, which revealed that incidences of HCC were still detected in the study as measured by ag-ELISA and there was no significant difference in cumulative incidences between the control and intervention villages, meaning that the intervention did not reduce incidences of HCC as hypothesized. Besides this study, no other study has estimated the cumulative incidence of HCC under field conditions. The intervention trial conducted in Mbulu District, northern Tanzania attempted to examine the incidence rate of PCC in sentinel pigs. The intervention was associated with a considerable decrease in the incidence rate of PCC (incidence rate ratio 0.57) as measured by antigen-ELISA in sentinel pigs. However, the findings from this previous study cannot be compared with the findings of the present study, because the previous study had some difficulty in controlling the experimental conditions and had other important limitations, including a short follow-up period of pigs (4 months), and the small sample sizes in many villages studied, which resulted from participants withdrawing from the study [13].

The aim of training the trainers, and distributing leaflets and booklets to the audience was to ensure the sustainability of the training even after the one-time health education session. Nevertheless, this might have increased the likelihood of contamination of the intervention in the control villages.

## 5. Conclusions

This study has shown that the adapted health-education intervention strategy is effective in increasing knowledge and improving pig-confinement practices against human TSTC transmission in selected villages in the Kongwa and Songwe Districts. This is an important step forward in the control of *T. solium* infections. The observed cumulative incidence of HCC is an indication of persisting environmental contamination with *T. solium* eggs and improper sanitary and hygienic practices, and, therefore, a high risk of HCC. This study recommends further studies to determine the effect of time as a factor on health-education interventions for improving preventive practices against human TSTC transmission and reducing incidences of HCC and other public health challenges.

## Figures and Tables

**Table 1:** Socio-demographic, behavioural and clinical characteristics at baseline (N = 662).

*Characteristic*	*Control (%)* *(n = 324)*	Intervention (%) (*n* = 338)	Chi Test	*p*-Value
**Socio-demographic**				
Sex			2.644	0.104
Male	230 (71.0)	220 (65.1)		
Female	94 (29.0)	118 (34.9)		
Age (years)			4.797	0.091
15–25	15 (4.6)	12 (3.6)		
26–45	131 (40.4)	165 (48.8)		
>45	178 (55.0)	161 (47.6)		
Educational level			1.156	0.764
Informal	250 (77.2)	255 (75.4)		
Primary	24 (7.4)	31 (9.2)		
Secondary	7 (2.2)	5 (1.5)		
Post-Secondary	43 (13.2)	47 (13.9)		
**Knowledge**				
Awareness of Human TSTC			0.340	0.560
No	283 (87.4)	290 (85.8)		
Yes	41 (12.6)	48 (14.2)		
Human consumption of PCC-infected pork is safe			0.121	0.728
No	30 (9.3)	34 (10.1)		
Yes	294 (90.7)	304 (89.9)		
*T. solium* infect both human and pigs.			2.294	0.130
No	306 (94.4)	309 (91.4)		
Yes	18 (5.6)	29 (8.6)		
Human TSTC be prevented			0.361	0.547
No	219 (67.6)	221 (65.4)		
Yes	105 (32.4)	117 (34.6)		
Human TSTC can cause epilepsy/seizures.			3.386	0.066
No	313 (96.6)	316 (93.5)		
Yes	11 (3.4)	22 (6.5)		
**Practice**				
Pigs roam freely in open fields			0.497	0.481
Closed system	6 (1.9)	4 (1.2)		
Freely roaming	318 (98.1)	334 (98.8)		
Always observe human faeces around the premises			0.140	0.708
No	160 (49.4)	162 (47.9)		
Yes	164 (50.6)	176 (52.1)		
Presence of toilet in household			0.459	0.498
No	132 (40.7)	129 (38.2)		
Yes	192 (59.3)	209 (61.8)		
Washing hands with soap after defecation			0.220	0.639
No	99 (30.6)	109 (32.3)		
Yes	225 (69.4)	229 (67.8)		
Use official water sources			0.045	0.832
No	260 (80.2)	269 (79.6)		
Yes	64 (19.8)	69 (20.4)		

**Table 2:** Post-intervention knowledge and practice related to human TSTC transmission between the intervention and control groups (N = 662).

Characteristic	Control (%)	Intervention (%)	Chi Test	*p*-Value
**Knowledge**				
Awareness of Human TSTC			123.895	<0.001
No	264 (81.5)	132 (39.1)		
Yes	60 (18.5)	206 (60.9)		
Human consumption of PCC-infected pork is safe			0.0001	0.976
No	323 (99.7)	337 (99.7)		
Yes	1 (0.3)	1 (0.3)		
*T. solium* infect both human and pigs.			24.600	<0.001
No	273 (84.3)	229 (67.8)		
Yes	51 (15.7)	109 (32.2)		
Human TSTC be prevented			84.035	<0.001
No	204 (63.0)	93 (27.5)		
Yes	120 (37.0)	245 (72.5)		
Human TSTC can cause epilepsy/seizures.			68.415	<0.001
No	266 (82.6)	174 (52.3)		
Yes	56 (17.4)	159 (47.8)		
**Practise**				
Pigs roam freely in open fields			11.42	<0.001
Closed system	172 (53.1)	223 (66.0)		
Freely roaming	152 (46.9)	115 (34.0)		
Always observe human faeces around the premises			3.305	0.069
No	100 (30.9)	127 (37.6)		
Yes	224 (69.1)	211 (62.4)		
Presence of toilet in household			2.985	0.084
No	178 (54.9)	163 (48.2)		
Yes	146 (45.1)	175 (51.8)		
Washing hands with soap after defecation			8.835	0.003
No	146 (45.)	115 (34.0)		
Yes	176 (54.7)	223 (66.0)		
Use official water sources			9.562	0.002
No	249 (76.9)	223 (66.0)		
Yes	75 (23.1)	115 (34.0)		

Significant at *p* < 0.05.

**Table 3:** Comparison of mean scores before and after the intervention between the Control and Intervention groups (N = 662).

Variable	Group	Pre-Intervention Mean ± SD	*p*-Value	Post-Intervention Mean ± SD	*p*-Value
Knowledge	Control	1.45 ± 0.94	0.235	0.94 ± 1.18	<0.001
	Intervention	1.54 ± 1.02		2.06 ± 1.45	
Practice	Control	2.98 ± 1.01	0.678	2.40 ± 1.13	0.306
	Intervention	3.01 ± 0.06		2.49 ± 1.13	

Upon the Sharpiro–Wilk test, the pre-intervention scores were not normally distributed but post-intervention scores were normally distributed. A *t*-test and Wilcoxon signed-rank test were used for comparison (due to the central limit theorem, with a sample size of more than 30, a paired *t*-test can also be used) and the *p*-value remained similar in each. Significance set at *p* < 0.05.

**Table 4:** Prevalence of HCC before and cumulative incidence after the intervention in the control and intervention villages.

Group	Pre-Intervention			Post-Intervention		
	(*n* = 872)	Prevalence (%)	*p*-Value	(*n* = 662)	Incidence (%)	*p*-Value
Control	432	6 (1.4)	0.97	324	6 (1.9)	0.48
Intervention	440	6 (1.4)		338	4 (1.2)	

## Data Availability

The datasets generated and analysed for this study are not publicly available due to participants’ privacy, but are available from the corresponding author upon reasonable request.
